# Phosphorylation as a regulatory mechanism of HP1 protein multifunctionality

**DOI:** 10.1007/s00412-025-00838-0

**Published:** 2025-10-15

**Authors:** James C. Walts, Nicole C. Riddle

**Affiliations:** Department of Biology, University of Alabama at Birmingham, Birmingham, AL USA

**Keywords:** Chromatin, Heterochromatin, Post-translational modifications, Gene regulation, HP1, Drosophila

## Abstract

The Heterochromatin Protein 1 (HP1) family proteins are key regulators of chromatin structure and genome function, acting as “reader” proteins that recognize and bind to histone H3 lysine 9 methylation (H3K9me). Beyond their canonical role in heterochromatin formation and transcriptional repression, HP1 proteins exhibit functional versatility, participating in transcriptional activation, RNA processing, DNA repair, and chromosome segregation. This multifunctionality is mediated partially by post-translational modifications (PTMs), with phosphorylation emerging as a central regulatory mechanism. This review explores the diverse effects of HP1 phosphorylation on protein function and chromatin interactions, focusing on *Drosophila melanogaster* HP1a and its orthologs, mammalian HP1α and *S. pombe* Swi6. Phosphorylation in the N-terminal tail enhances HP1’s affinity for H3K9me, promoting transcriptional silencing. Mitotic phosphorylation of serine residues in the hinge region, regulated by kinases such as AURKB and NDR1/2, leads to chromatin release and relocalization to the kinetochore, enabling proper chromosome segregation. Additionally, phosphorylation modulates HP1 phase separation dynamics, influencing nuclear compartmentalization and chromatin condensation. These findings highlight phosphorylation as a versatile molecular switch that enables HP1 proteins to transition between structural and regulatory roles, contributing to their evolutionary conserved multifunctionality in genome regulation and cell division. Further investigation into HP1 phosphorylation across species and contexts is essential to fully understand its contributions to chromatin biology.

**Supplementary Information:**

The online version contains supplementary material available at 10.1007/s00412-025-00838-0.

## Introduction

In eukaryotes, chromatin functions to ensure DNA integrity and regulate transcription (Cramer 2019). An octamer of four histone proteins – two copies each of H2A, H2B, H3, and H4 – and 147 bp of DNA comprise the main unit of chromatin, the nucleosome (Kornberg 1974; Luger et al. 1997). The disordered N-terminal tails of these histone proteins are post-translationally modified through, for example, methylation, acetylation, and phosphorylation (Jenuwein and Allis 2001; Tessarz and Kouzarides 2014; Taylor and Young 2021; Liu and Schneider 2025). These post-translational modifications (PTMs) are regulated and utilized by non-histone chromosomal proteins, or proteins of diverse functions that can associate with chromatin (Cartwright et al. 1982). Non-histone chromosomal proteins often are classified into three roles: “writer” proteins add PTMs to histones, “eraser” proteins remove PTMs from histones, and “reader” proteins recognize and bind to certain PTMs. The Heterochromatin Protein 1 (HP1) family, are non-histone chromosomal proteins that acts as “reader” proteins, typically recognizing methylated lysine 9 on histone H3 (H3K9me) (Bannister et al. 2001; Lachner et al. 2001). Together, modifications on histones and non-histone chromosomal proteins specify chromatin structure, regulate access, and determine transcriptional state.

**Fig. 1:**
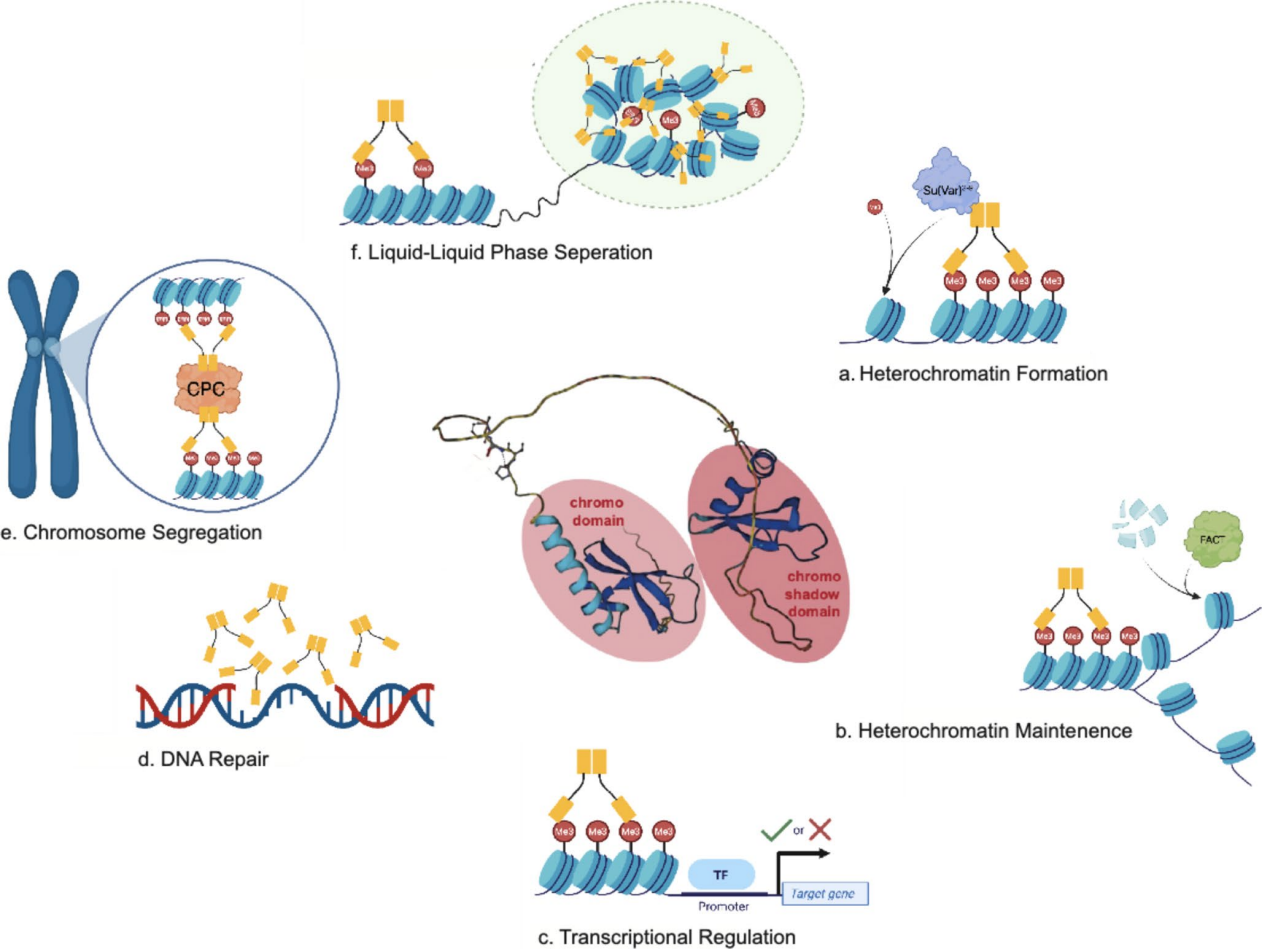
Schematic representation of the multiple functions of HP1 proteins. HP1 proteins (middle, predicted alpha fold model) are comprised of two structured regions, the chromodomain and the chromo-shadow domain. Alongside their binding partners HP1 proteins (yellow) aid in heterochromatin formation (**a**) and maintenance (**b**), transcriptional regulation (**c**), DNA repair (**d**), chromosome segregation (**e**), and liquid-liquid phase separation (**f**). Created in BioRender

HP1 proteins are integral to the formation and maintenance of highly condensed and transcriptionally repressed heterochromatin, a specialized form of chromatin often associated with centromeres and telomeres enriched in di- and trimethylated histone 3 lysine 9 (H3K9me2/me3) (Grewal and Jia 2007; Allshire and Madhani 2018; Grewal 2023). Loss of HP1 proteins impairs heterochromatin structure and function, which in *D. melanogaster* and *S. pombe* leads to a loss of heterochromatin structures (Eissenberg et al. 1990, 1992; Lorentz et al. 1994; Lu et al. 2000), while in mouse, DAPI-dense chromocenters remain even in the absence of HP1 proteins and H3K9 methylation and the presence of other heterochromatin defects (Peters et al. 2001; Velichko et al. 2011; Bosch-Presegué et al. 2017; Saksouk et al. 2020; Dupont et al. 2023). All HP1 proteins share a common domain structure. They have an N-terminal chromodomain (CD), a C-terminal chromo-shadow domain (CSD), and a variable hinge region that connects the conserved CD and CSD (Paro and Hogness 1991; Assland and Stewart 1995) (Fig. 1). Some HP1 proteins also have N- and C-terminal tails of varying lengths. The CD is responsible for recognition and binding to H3K9me2/3 (Paro and Hogness 1991; Bannister et al. 2001; Lachner et al. 2001; Nielsen et al. 2002), while the CSD mediates dimerization of HP1 proteins (Assland and Stewart 1995; Cowieson et al. 2000; Smothers and Henikoff 2000). In addition, the CSD also mediates interactions of dimerized HP1 proteins with other protein partners (Assland and Stewart 1995; Cowieson et al. 2000; Smothers and Henikoff 2000). The hinge region can bind nucleic acids (Meehan 2003; Tachiwana and Saitoh 2025) (Fig. 1). Thus, the CD, hinge, and CSD have distinct roles that contribute to the function of HP1 proteins in heterochromatin formation and maintenance.

The properties of HP1’s CD and CSD are important for the ability of heterochromatin to form large blocks of this unique chromatin structure. Once initiated at a specific site in the genome, heterochromatin can spread along the DNA. This spreading is facilitated by the interaction of HP1 proteins with both H3K9 methylation and the enzymes producing this modification (Grewal and Jia 2007; Allshire and Madhani 2018). For example, the *Drosophila melanogaster* HP1 protein HP1a binds to H3K9me2/3 through its CD (Paro and Hogness 1991; Bannister et al. 2001; Lachner et al. 2001; Nielsen et al. 2002). HP1a also interacts with the H3K9 histone methyltransferase (HMT) Su(var)3–9 via its CSD (Aagaard 1999; Schotta 2002). Thus, HP1a recruits Su(var)3–9 to areas that already have some H3K9 methylation, Su(var)3–9 then methylates nearby H3K9, which in turn increases the number of HP1a binding sites (Bannister et al. 2001; Lachner et al. 2001; Nakayama et al. 2001; Ebert et al. 2006) (Fig. 1a). Association with heterochromatin through binding to H3K9 methylation and recruiting HMTs is an evolutionarily conserved function with Swi6 (yeast) and HP1α (mammalian) orthologs also having this function (Bannister et al. 2001; Grewal and Elgin 2002; Krouwels et al. 2005). This cycle is able to propagate heterochromatin along the chromatin fiber, leading to the formation of long tracts of heterochromatin, for example at the centromeres and telomeres (Pidoux and Allshire 2005; Chow et al. 2018; Schoelz and Riddle 2022).

Moonlighting proteins (MLPs) are a subgroup of multifunctional proteins encoded by one gene that, under different conditions, can perform multiple functions regulated independently of one another (Jeffery 1999, 2016, 2018; Henderson and Martin 2014). Interestingly, despite being named for their function in heterochromatin maintenance, HP1 proteins have other functions as well. HP1a is a MLP that performs several duties – transcriptional activation and elongation, sister chromatid cohesion, chromosome segregation, telomere maintenance, DNA repair, and RNA splicing – outside of its primary function, transcriptional silencing (Fanti and Pimpinelli 2008; Piacentini et al. 2009; Vermaak and Malik 2009; Chiolo et al. 2011; Schoelz and Riddle 2022) (Fig. 1). For example, the mammalian HP1 proteins (HP1α, HP1β, and HP1γ) all are recruited to sites of both ultraviolet (UV) lesions and DNA double strand breaks (DSBs) independent of the known DNA damage recognition proteins (Luijsterburg et al. 2009) (Fig. 1d). According to one model, the protein ACF1 recruits HP1 proteins to these sites of DNA damage (Eskeland et al. 2007). ACF1 interacts with the CSD of HP1 proteins and accumulates at the site of UV lesions (Eskeland et al. 2007). ACF1 may cooperate with mammalian HP1 proteins to modify the chromatin landscape at the site of damaged DNA, creating a more “open” chromatin environment for DNA repair factors (Eskeland et al. 2007). Additionally, nematodes deficient in Heterochromatin Protein Like (HPL), the *Caenorhabditis elegans* HP1 homolog, are especially sensitive to UV-induced DNA damage, suggesting HPL’s importance in its repair and providing another example of a role of a HP1 protein beyond pathways for the formation and maintenance of heterochromatin (Luijsterburg et al. 2009).

Several hypotheses have been proposed to explain how the HP1 family of proteins can carry out the many functions that have been described for them. First, by the fact that many eukaryotes have more than one HP1 paralog (*Saccharomyces pombe* Chp2 and Swi6; *Drosophila melanogaster* HP1a, HP1B, HP1C, Rhino, HP1E; *Mus musculus*/*Homo sapiens* HP1α, HP1β, HP1γ, or CBX5, CBX1, and CBX3), each of which likely evolved to carry out specific functions (Lomberk et al. 2006). Second, through their CSD HP1 proteins can dimerize and recruit binding partners containing PxVxL–like sequences (PGTVAL in the αN1-helix of histone H3, LSVKI of *D. melanogaster* HP2 protein, PRVKV of *D. melanogaster* PIWI protein, or PVVVL of *M. musculus* CAF-1 p150 subunit) (Smothers and Henikoff 2000; Thiru et al. 2004; Dawson et al. 2009; Lavigne et al. 2009; Mendez et al. 2011, 2013; Richart et al. 2012). Lastly, similar to histones, HP1 proteins also carry PTMs and can be modified by methylation, acetylation, phosphorylation, SUMOylation, ubiquitination, and citrullination (Sales-Gil and Vagnarelli 2020). Compared to HP1 paralogs and binding partners, we know substantially less about the molecular impacts HP1 modifications have on HP1 functions.

In this review, we will use *Drosophila melanogaster* HP1 proteins as a model, supplemented with data from other species, to explore the role of HP1 phosphorylation, its regulation, and the functional consequences on chromatin dynamics and cellular processes. We will discuss the various enzymes involved in regulating phosphorylation, the specific sites of phosphorylation, their functions, and how these modifications affect interactions with chromatin and protein partners. There is limited understanding of how altering phosphorylation of HP1 proteins impacts their functions and thus how it impacts the organism’s health and survival. This review aims to provide relevant information on how phosphorylation impacts the multifunctionality of HP1 proteins, with a particular focus on *D. melanogaster* HP1a and its orthologs, HP1α and Swi6, and their functions in heterochromatin formation and maintenance as well as during cell division.

## Post-translational modifications and their role in eukaryotes

PTMs have the potential to greatly increase the complexity of proteomes. Due to PTMs, the proteome of an organism can be two to three orders of magnitude larger than predicted by genome annotations (Walsh 2006). For example, the human genome is estimated to contain 30,000 genes, yet the human proteome is estimated to consist of 300,000 to 3,000,000 distinct protein isoforms (Walsh 2006). While alternative splicing contributes to this increased proteome diversity (Maniatis and Tasic 2002; Black 2003), a large part of this diversity is due to PTMs. PTMs are generated by proteolytic cleavage or by the addition of a modifying group, such as acetyl-, phosphoryl-, glycosyl- and methyl-groups, to one or more amino acids (Walsh 2006). PTMs regulate protein activity, localization, and interaction with other cellular molecules (Duan and Walther 2015). HP1 proteins typically only are targeted by reversible PTMs.

Of the nearly 200 types of reversible PTMs that occur by covalent addition of side chains nearly all are carried out by enzymes (http://www.uniprot.org/docs/ptmlist.txt). For phosphorylation alone, in humans, there are more than 650 enzymes involved in regulating this modification (500 kinases and 150 phosphatases) (Walsh 2006). Kinases phosphorylate proteins, and phosphatases remove phosphate groups, creating a reversible process that allows proteins to switch between states and sometimes functional roles (Cohen 2002). Kinases phosphorylate serine, threonine, and tyrosine (histidine and aspartic acid in bacteria and fungi) (Mann et al. 2002). Instead of phosphorylating specific proteins, kinases tend to phosphorylate one to two amino acid residues (Shi et al. 1998). For example, casein kinase II and the cAMP-dependent protein kinase both phosphorylate serine/threonine residues, while the insulin receptor kinase and pp60^src^ phosphorylate tyrosine residues (Shi et al. 1998). This “promiscuity” of kinases makes it difficult to study the consequences of phosphorylation of individual proteins by blocking kinases, a challenge for the study of HP1 PTMs. In addition to the phosphorylated residue, phosphatase specificity can be influenced by the surrounding amino acid residues and the active site of the phosphatase (Jeffrey et al. 2007). Substantially less is known about the phosphatases involved in HP1 PTM regulation compared to HP1 kinases.

**Fig. 2:**
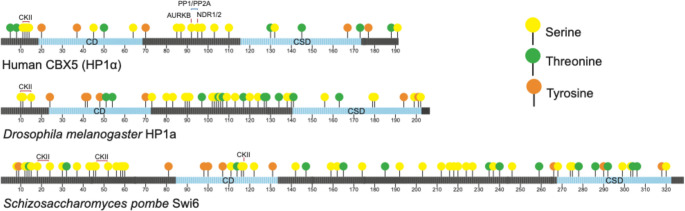
HP1 proteins contain many amino acid residues capable of being phosphorylated. HP1 proteins contain multiple serine (yellow), threonine (green), and tyrosine (orange) residues capable of being phosphorylated. Human HP1α (CBX5) (top) contains 15 serine, 7 threonine, and 5 tyrosine residues. *Drosophila melanogaster* HP1a (middle) contains 24 serine, 11 threonine, and 7 tyrosine residues. *Schizosaccharomyces pombe* Swi6 (bottom) contains 40 serine, 15 threonine, and 9 tyrosine residues. CKII, AURKB, and NDR1/2 kinases are known to phosphorylate serine residues (denoted by the red lines) found within the NTE and hinge region. PP1/PP2a phosphatase is known to remove hinge region phosphorylation (denoted by the blue lines). For additional details see Table S1

Phosphorylation is the most abundant and well-studied of HP1’s PTMs, and several studies have identified specific phosphorylation sites and the enzymes involved in regulating the phosphorylation. HP1 proteins can be multiply phosphorylated, with the phosphorylation taking place at serine, threonine, and tyrosine residues (Eissenberg et al. 1994). *D. melanogaster* HP1a, mammalian HP1α, and *S. pombe* Swi6 contain 27, 42, and 64 amino acid residues, respectively, that could be phosphorylated, with only a few residues having been tested experimentally (Fig. 2; Table 1). For example, mammalian HP1 proteins - HP1α, HP1β, and HP1γ - contain 17, 16, and 16 serine, threonine, and tyrosine residues respectively capable of being phosphorylated (LeRoy et al. 2009; Sales-Gil and Vagnarelli 2020). Eissenberg and colleagues were able to define eight consensus target sites for kinases on *D. melanogaster* HP1a in embryo-derived tissue culture cells (Zhao et al. 2001). Using protein samples from larvae (salivary gland, fat body, imaginal discs, larval brain), they detected six to eight HP1a phospho-isoforms (Minc et al. 1999; Zhao et al. 2001). Further studies using mutants either blocking or mimicking phosphorylation have begun to reveal the functional roles that phosphorylation impacts, but these studies largely focus on multiple phosphorylation sites generating a broad understanding of how phosphorylation contributes to the multifunctionality of HP1 proteins (Zhao et al. 2001; Badugu et al. 2005).

**Table 1:** Domain architecture and length for major HP1 orthologs. Human HP1α (CBX5) (first row) has a total length of 191 amino acids (aa), *Drosophila melanogaster* HP1a (second row) has a total length of 206 aa, and *Schizosaccharomyces Pombe* Swi6 (third row) has a total length of 328 aa. For each protein aa range (top), domain length (middle), and number of aa that can be phosphorylated are listed (bottom). Based on information retrieved from NCBI (protein identifiers are listed in column 2). NTE: N-terminal extension; CD: Chromodomain; CSD: Chromo shadow domain; CTE: C-terminal extension

Protein	NCBI ID	Total Length (aa)	NTE (aa)	CD (aa)	Hinge (aa)	CSD (aa)	CTE (aa)
Human HP1α (CBX5)	23468	191	1–18 (18) 6	19–68 (50) 5	69–115 (47) 8	116–173 (58) 5	174–191 (18) 3
*D. mel* HP1a	34119	206	1–23 (23) 3	24–72 (49) 7	73–140 (68) 23	141–202 (62) 9	203–206 (4) 0
*S. pombe* Swi6	2541633	328	1–84 (84) 20	85–133 (49) 9	134–267 (134) 21	268–322 (55) 13	323–328 (6) 0

## Transcriptional regulation and heterochromatin maintenance

*D. melanogaster* HP1a was the first characterized HP1 protein (James and Elgin 1986). It was identified using monoclonal antibodies against proteins that had been isolated from embryo nuclei and further characterized by its molecular weight, localization to heterochromatin, and cDNA (James and Elgin 1986). Subsequent studies using a variety of techniques (tethering (Lachner et al. 2001; Nakayama et al. 2001), PEV (Reuter et al. 1986; Eissenberg et al. 1990, 1992; Cryderman et al. 1998; Elgin and Reuter 2013), and gene expression analysis (Cryderman et al. 2005; De Lucia et al. 2005; Riddle et al. 2012; Lee et al. 2013; Park et al. 2019) identified HP1a as a repressor of transcription through its ability to bind to and propagate the heterochromatic H3K9me2/3 histone mark (Grewal and Jia 2007; Allshire and Madhani 2018). However, these same assays showed that under certain conditions HP1a acts as an activator of transcription (Wakimoto and Hearn 1990; Lu et al. 2000; Schulze et al. 2005; Riddle et al. 2012). Genes found in the pericentric heterochromatin experience the opposite effect of their euchromatic gene counterparts, with HP1a being required for transcription (Wakimoto and Hearn 1990; Lu et al. 2000; Schulze et al. 2005; Riddle et al. 2012). Transcriptional regulation and heterochromatin maintenance are the best characterized functions of HP1a, but few studies have investigated how phosphorylation impacts these roles of HP1a.

### Transcriptional regulation

As noted above, HP1a was described initially as a transcriptional repressor due to its localization to areas of heterochromatin – pericentric heterochromatin, telomeres, and chromosome 4 in *D. melanogaster* – and as a modifier of position effect variegation (PEV) (James et al. 1989). In PEV assays, HP1a acts in a dosage-dependent manner to suppress variegation (Elgin and Reuter 2013). Using an X chromosome inversion of the *white* gene (*In(1)w*^*m4*^), Elgin and Reuter tested how the amount of HP1a available affected PEV (Elgin and Reuter 2013). They showed that heterozygous individuals, with one functional copy of HP1a, had increased expression of *white* and less heterochromatin formation, while animals with three functional copies of HP1a had decreased expression of *white* and more heterochromatin formation (Elgin and Reuter 2013). Follow-up studies showed that when the *white* is moved close to heterochromatin, it gains H3K9 methylation, mediated by HP1a (Li et al. 2003; Vogel et al. 2009; Elgin and Reuter 2013). HP1a’s role as a transcriptional repressor also can be seen using tethering studies, where HP1a-lacI fusion proteins were recruited to promoters with *lacO* repeats (Li et al. 2003). Using this method, Wallrath and colleagues observed repression of the *white* gene at 25 of 26 genomic locations (Li et al. 2003). Together, these studies support HP1a’s role as a transcriptional repressor.

When examining genes normally found within the heterochromatin, early studies found that HP1a has the opposite effect on these genes – the presence of HP1a aids in expression of these genes (Wakimoto and Hearn 1990; Lu et al. 2000). For example, *rolled* encoding a *D. melanogaster* ERK-1/MAP kinase is located deep within the pericentric heterochromatin of the right arm of chromosome 2 (Hilliker and Holm 1975). Using rearrangements that placed *rolled* within euchromatin, Elgin and colleagues found that HP1a is a significant enhancer of *rolled* position effect, a finding that demonstrates that the presence of HP1a is required for the expression of *rolled* (Hilliker and Holm 1975). Additionally, the *D. melanogaster light* gene is located within or near the centromeric heterochromatin on the left arm of chromosome 2 (Wakimoto and Hearn 1990). Wakimoto and colleagues used chromosomal rearrangements to place the *light* gene within distal euchromatin, and this rearrangement resulted in variegated expression of the *light* gene (Wakimoto and Hearn 1990). In addition to looking at the *light* gene they also observed the effect of this rearrangement on seven heterochromatic genes that normally neighbor the *light* gene (Wakimoto and Hearn 1990). They showed that proximity to distal euchromatin inactivated five of the seven neighboring genes (Wakimoto and Hearn 1990). Together, these experiments manipulating the amount of HP1a and observing the effect on reporter genes such as *white*, *rolled*, or *light* inserted at strategic locations in the genome demonstrate that HP1a can act as both a suppressor and enhancer of variegation. Depending on the genome context, HP1a plays an opposing role in transcriptional regulation, acting as a repressor when associated with euchromatic genes relocated to heterochromatin, and as an activator for heterochromatic genes, highlighting its complex impact on gene expression.

Genome-wide transcription studies support the results from PEV studies. Gene expression changes following the loss of HP1a show that over half of the misregulated genes are downregulated. For example, studies using RNA-seq found that 60% of misregulated genes were downregulated (Riddle et al. 2012), and in stage 14 eggs from animals depleted for HP1a by RNAi 623 upregulated and 736 downregulated genes were found (Park et al. 2019). Riddle and colleagues analyzed three RNA-seq datasets using HP1 knockout (KO) mutants and found that ~ 49% of HP1a bound genes were differentially expressed but that only ~ 20% of the differentially expressed genes were binding targets, suggesting a majority of the expression changes observed are indirect effects of the loss of HP1a (Riddle et al. 2012; Mills et al. 2018; Schoelz et al. 2021). While these indirect effects make interpretation of transcriptome changes more challenging, these studies consistently showed that transposable elements (TEs) are upregulated in HP1a KO mutants (Vermaak and Malik 2009; Riddle et al. 2012; Mills et al. 2018; Schoelz et al. 2021). Misregulation of TEs can lead to abnormal development, increased disease susceptibility, changes in gene expression patterns, altered morphology, reduced fertility, and even accelerated aging (Rigal et al. 2022; Copley and Shorter 2023). These findings show the widespread impact of HP1a on genome stability and transcriptional regulation, highlighting its essential role in maintaining proper gene expression and suppressing TE activity.

Despite the importance of HP1a in the regulation of TEs and gene expression, only a handful of studies have investigated how phosphorylation of HP1a proteins impacts its role as a transcriptional regulator (Zhao and Eissenberg 1999; Shimada et al. 2009; Hiragami-Hamada et al. 2011; Nishibuchi et al. 2014; Shimojo et al. 2016). Casein Kinase II (CKII) phosphorylates serines in the N-terminal and C-terminal tails of three HP1a homologs - HP1a, Swi6, and HP1α (Zhao and Eissenberg 1999; Shimada et al. 2009; Hiragami-Hamada et al. 2011; Nishibuchi et al. 2014; Shimojo et al. 2016). Phosphorylation of serines within the N-terminal extension (NTE) of HP1a by CKII leads to increased recognition and binding to H3K9me2/3 (Zhao and Eissenberg 1999; Shimada et al. 2009; Hiragami-Hamada et al. 2011; Nishibuchi et al. 2014; Shimojo et al. 2016). Phospho-mimic mutants substituting serine to glutamate (S-E) recapitulate the enhanced binding of HP1a’s CD to H3K9 methylation, and phospho-block mutants substituting serine to alanine (S-A) result in a loss of genomic silencing (Hiragami-Hamada et al. 2011). Further study of the NTE region of HP1a and other HP1 proteins reveal crucial differences in the amino acid composition of the NTE and its affinity to H3K9me3. For example, when comparing mammalian HP1 paralogs, HP1β and HP1γ have glutamate residues, a negatively charged amino acid, instead of serine residues in their NTE. This difference results in their disassociation constant (K_D_) for H3K9me3 tails to be 5.8 µM and 12 µM, respectively, while unphosphorylated HP1α has a binding affinity of 35 µM (Hiragami-Hamada et al. 2011). When phosphorylated by CKII at the NTE serine residues, H3K9me3 binding by HP1α increases nearly fivefold to a K_D_ of 8.3 µM (Hiragami-Hamada et al. 2011). Like mammalian HP1β and HP1γ, Swi6, one of two *S. pombe* HP1 proteins, does not contain serine residues in the NTE, but rather has several acidic residues. In 2016 Shimojo and colleagues examined how phosphorylation of the serine residue within the NTE of HP1α results in greater binding affinity with H3K9me3 tails. Using nuclear magnetic resonance (NMR), small angle X-ray scattering, and systematic analysis of NTE deletion mutants, they determined that phosphorylation of HP1α within the NTE modulated the flexibility of the N-terminal tail, but does not impact the structure of the aromatic cage that serves as binding pocket for the H3K9me2/3 peptide (Shimojo et al. 2016) [the lack of change to the aromatic cage was confirmed more recently for other reader proteins by Kamps and colleagues as well] (Kamps et al. 2015). In these studies, the phosphorylated NTE of HP1α takes on a more extended conformation, and there are increased interactions between the phosphorylated serines in the HP1α NTE and a basic patch of lysines present in the H3 peptide. A 2025 follow-up study from the same laboratory confirms this finding and finds that NTE phosphorylation of HP1α also leads to increased interactions with other basic patches in the HP1α hinge region and increases interactions between HP1α molecules, via the same basic patches and via the CD (Furukawa et al. 2025). Thus, the available data suggest that at least some phosphorylation sites, particularly those in the NTE, can impact the ability of HP1 proteins to interact with repressive chromatin marks. These findings highlight how phosphorylation directly influences HP1a’s ability to bind repressive chromatin marks, thereby modulating its function in the transcriptional silencing of target genes.

### Heterochromatin formation and maintenance

As noted earlier, many HP1 proteins function in the establishment and maintenance of heterochromatin. Nearly one-third of the *D. melanogaster* genome, including the telomeres, pericentric regions, centromeres, and chromosomes 4 and Y, is comprised of heterochromatin (Adams et al. 2000). There are two types of heterochromatin: constitutive heterochromatin and facultative heterochromatin. Constitutive heterochromatin is formed early in eukaryotic development and remains condensed and transcriptionally inactive in all cells (Saksouk et al. 2015; Grewal 2023). Constitutive heterochromatin typically is found in regions containing repetitive DNA sequences - the centromeres and telomeres (Saksouk et al. 2015; Grewal 2023). As the organism develops, and cells differentiate, facultative heterochromatin is formed over various sections of the genome to prevent inappropriate gene expression (Saksouk et al. 2015; Grewal 2023). *D. melanogaster* HP1a is found at both regions of constitutive and facultative heterochromatin, and together with H3K9me2/3 is what defines this specialized chromatin type (Saksouk et al. 2015; Grewal 2023).

In the nucleus, heterochromatin tends to form a unique compartment, and HP1 proteins are important for this compartmentalization. Heterochromatin often is located at the nuclear lamina, a protein network at the inner surface of the nuclear envelope (Akhtar and Gasser 2007; Steensel and Belmont 2017), with genes located at the periphery usually being silenced (Andrulis et al. 1998; Finlan et al. 2008; Reddy et al. 2008; Robson et al. 2016). In the fission yeast *S. pombe*, the HP1 protein Swi6 works together with Amo1, the Rix1 complex (RIXC), and Facilitates Chromatin Transactions (FACT) complex to organize heterochromatin at the nuclear lamina, establish heterochromatin domains, and maintain heterochromatin through replication and transcription events (Holla et al. 2020). Swi6, through its CD, binds to H3K9me2/3 and, through its CSD, interacts with RIXC (Holla et al. 2020). RIXC then acts as a bridge, connecting heterochromatin domains to distinct pools of Amo1 located in the nuclear envelope (Holla et al. 2020). In addition to anchoring heterochromatin to the nuclear envelope, Amo1 also recruits additional factors required for the propagation of heterochromatin such as FACT (Holla et al. 2020). During transcription and DNA replication, FACT associates with Swi6 and Amo1 to promote epigenic stability by repressing histone turnover through retention of pre-existing histones (Belotserkovskaya et al. 2003; Jamai et al. 2009; Svensson et al. 2015). Since Amo1 associates with FACT and other repressive factors, it is thought that at these pools of Amo1 within the nuclear envelope there are specialized subdomains with high concentrations of these silencing factors. This example illustrates how heterochromatin enriched for an HP1 protein can facilitate the formation of specific nuclear compartments.

Liquid-liquid phase separation (LLPS) further contributes to the compartmentalization of HP1 proteins and heterochromatin in the nucleus (Larson et al. 2017; Strom et al. 2017; Larson and Narlikar 2018; Sanulli and Narlikar 2020; Keenen et al. 2021; Tortora et al. 2023; Brennan et al. 2024). HP1 proteins, as well as many other proteins, including Amo1, have the ability to form phase-separated liquid droplets, suggesting that these proteins can create a compartment of silencing factors through LLPS in the nucleus (Frey et al. 2006; Larson et al. 2017; Strom et al. 2017). While the ability to undergo LLPS is dependent on the protein, particular the presence of intrinsically disordered regions, it also is highly context-dependent, with LLPS occurring at specific concentrations of proteins, dependent on salt concentration, as well as the amount of other proteins or DNAs present. The ability of HP1 proteins to undergo LLPS has been studied in vitro and in vivo for HP1a (in vivo), HP1α (in vivo and in vitro), and Swi6 (in vivo) (Larson et al. 2017; Strom et al. 2017; Sanulli et al. 2019; Erdel et al. 2020; Bensaha et al. 2025), and several factors that affect LLPS of these HP1 proteins have been identified. Phosphorylation of a serine patch located in the NTE region of some HP1 proteins contributes to LLPS (Larson et al. 2017; Her et al. 2022), as do multivalent interactions of HP1 proteins with DNA and chromatin (Larson et al. 2017; Wang et al. 2019; Keenen et al. 2021). Ligands that target a specific binding site located at the HP1 CSD–CSD dimer interface also can contribute to LLPS of HP1 proteins (Larson et al. 2017; Wang et al. 2019; Keenen et al. 2021). These three aspects of HP1 biochemistry work together in complex ways to regulate LLPS of HP1 proteins.

Phosphorylation likely contributes to LLPS due to its impact on the overall charge of HP1 proteins. In the nuclear environment, HP1 proteins typically have a net negative charge, with only the disordered N-terminal tail and hinge region having a positive charge (Her et al. 2022). For example, the mammalian HP1α hinge region and N-terminal tail have a positive charge of + 6.9 and + 0.9, respectively (Her et al. 2022). Mammalian HP1α can be phosphorylated at serines S11-14, which drastically drops the charge of the N-terminal tail, creating a conformation that favors interactions between the N-terminal tail and the positively charged ‘KRK’ (amino acids 89–91) region in the hinge domain (Larson et al. 2017; Her et al. 2022). This interaction then lengthens the HP1α homodimer to allow each N-terminal tail to further interact with the hinge region of additional HP1α homodimers. While mammalian HP1α requires phosphorylation of these serine residues for efficient LLPS in vitro, unphosphorylated HP1α can undergo LLPS under specific conditions (Larson et al. 2017). Work from Furukawa and colleagues demonstrates that while HP1α phosphorylated at its NTE phase-separates more readily through the formation of HP1α multimers, unphosphorylated HP1α can still phase-separate, but via a different mechanism that relies on intra-molecular interactions (Furukawa et al. 2025). Phosphorylation is not required for the *D. melanogaster* HP1a or *S. pombe* Swi6 to undergo LLPS (Strom et al. 2017; Sanulli et al. 2019). Thus, while N-terminal tail phosphorylation is important for some HP1 proteins and can change LLPS dynamics, it is not the sole driver of LLPS for HP1 proteins.

Within the LLPS condensates, HP1 proteins can interact with DNA through their hinge regions to compact chromatin (Meehan 2003; Canzio et al. 2011; Mishima et al. 2013; Azzaz et al. 2014; Kilic et al. 2015, 2018). Based on work with HP1α, Keenan and colleagues suggested that this process could be regulated at three different stages (Keenen et al. 2021). First, before DNA condensation when HP1α is being loaded onto DNA. Second, when HP1α-DNA and HP1α-DNA interactions compact uncompacted DNA into the condensate. Third, when the condensate grows large enough to encounter remodeling forces either strengthening or weakening these condensates (Keenen et al. 2021). For example, Her and colleagues tested four peptides of either slightly stronger (Sgo1 and CAF1) or slightly weaker (LBR and H3) HP1α binding affinity (Her et al. 2022). These peptides were mixed with either HP1α alone or a HP1α-DNA mixture (Her et al. 2022). This experiment showed that while binding affinity did not play a role in LLPS, peptide charge did (Her et al. 2022). Sgo1 and H3, both positively charged, increased HP1α’s ability to complete LLPS, while CAF1 and LBR, both negatively charged, decreased it (Her et al. 2022). This example demonstrates how the charge of HP1 binding partners affects LLPS, but similar studies focused on alterations to the charge of HP1 proteins, altering negative charge through phosphorylation for example, are lacking. For *D. melanogaster* HP1a, how HP1a liquid droplets form, if charge alters LLPS dynamics, and how these droplets impact HP1a’s functions, primarily transcriptional regulation, mostly is unknown. It is also important to combine in vitro studies of LLPS with in vivo studies, as two recent publications suggest that mammalian HP1 proteins likely do not undergo LLPS in the nucleus under standard conditions (Erdel et al. 2020; Bensaha et al. 2025). Further studies are needed from a more diverse set of HP1 proteins to gain an in-depth understanding of how phosphorylation and potentially other PTMs impact the ability of HP1 proteins to participate in LLPS, especially in vivo.

## HP1a’s role in cellular division

HP1 proteins also have functions in cell division and chromosome segregation, and there is some evidence that phosphorylation impacts these functions of HP1 proteins as well. Throughout the cell cycle, the localization of HP1 proteins changes, with the majority of HP1 being removed from the chromosomes during mitosis and only small deposits being retained at the centromere. HP1 retention at the centromere is important for promoting the proper segregation of chromosomes in *D. melanogaster* embryos (Kellum and Alberts 1995), *S. pombe* (Ekwall et al. 1995; Nakayama et al. 2000), and in mammalian cells (Quivy et al. 2008; Kiyomitsu et al. 2010). For example, in *S. pombe*, Swi6 links heterochromatin and cohesion, ensuring sister-chromatid cohesion and correct chromosome segregation (Nonaka et al. 2002). This function is conserved across species, illustrated by the fact that mammalian HP1α acts in a dosage dependent manner at the centromere with overexpression and knockdown mutants resulting in cohesion defects (De Koning et al. 2009; Abe et al. 2016). The cell-cycle-dependent localization of HP1 and its association with cohesion at the centromere highlight a conserved function across many HP1 orthologs, where their presence at the centromere is necessary for ensuring correct chromosome segregation.

The cell-cycle dynamics of HP1 phosphorylation isoforms are best understood for HP1α, where immunofluorescence microscopy and biochemical assays were used to track its localization and phosphorylation state throughout the cell cycle (Minc et al. 1999). During interphase, two isoforms of HP1α associate with chromatin, but during mitosis, two additional, more acidic, isoforms of HP1α can be identified (Minc et al. 1999). Two amino acid residues, S92 and S95, are phosphorylated dynamically during the cell cycle and have been studied for their impact on HP1α’s role in the cell cycle (Chakraborty and Prasanth 2014; Chakraborty et al. 2014; Nishibuchi et al. 2019; Williams et al. 2019). Several other, less studied, residues S85, S87, S97, and S110 also have been identified as potential mitotic phosphorylation sites (Nishibuchi et al. 2019). All these amino acid residues are found within HP1α’s hinge region, within a basic patch of amino acid residue that regulates HP1α’s ability to bind to DNA, suggesting a connection between HP1α’s ability to bind to DNA and its role in the cell cycle (Meehan 2003; Mishima et al. 2013; Nishibuchi et al. 2014). Nishibuchi and colleagues found that during prophase HP1α S92 is phosphorylated by Aurora kinase B (AURKB) and results in a reduction of HP1α binding to both methylated and unmethylated DNA (Nishibuchi et al. 2019). Similarly, Chakraborty and colleagues showed that the nuclear Dbf2-related kinases (NDR1/2) phosphorylate HP1α at S95 during G2, again resulting in the detachment of HP1α from chromatin and its relocalization to the kinetochore (Chakraborty and Prasanth 2014; Chakraborty et al. 2014). Initially, it was thought that loss of HP1α binding of chromosomes during mitosis was due to changes in histone PTMs, specifically phosphorylation of H3S10 by AURKB, which prevents HP1α’s CD from binding to H3K9me (Fischle et al. 2005; Hirota et al. 2005). However, given the dynamic nature of HP1α phosphorylation, it is likely a combination of mitotic phosphorylation of HP1α’s hinge region and phosphorylation of H3S10 that removes HP1α from most chromatin and relocalizes it to the kinetochore (Nishibuchi et al. 2019). These findings from mammalian HP1α show that mitotic phosphorylation can regulate the function and localization of HP1 proteins in a cell-cycle-dependent manner.

Studies of mutant HP1α alleles with specific amino acid substitutions in the hinge domain provides additional insights into the impact of phosphorylation on HP1α function. Loss of phosphorylation using phospho-block (S92A or S95A) mutants results in mitotic chromosome instability, anaphase/telophase chromatin bridges, micronuclei formation, and aberrant spindle morphology (Chakraborty and Prasanth 2014; Chakraborty et al. 2014; Nishibuchi et al. 2019). Additionally, S95A mutant HP1α failed to localize to the kinetochore, suggesting that phosphorylation is necessary for HP1α to interact with its binding partner Sgo1, which protects centromeric sister chromatid cohesion during prophase (Kitajima et al. 2004; Chakraborty et al. 2014). Phospho-mimic (S92D and S95E) mutant HP1α or wildtype HP1α are able to rescue spindle assembly and micronuclei formation (Chakraborty et al. 2014; Williams et al. 2019). These phospho-mimic mutants also result in the accumulation of cells in telophase, suggesting that dephosphorylation of HP1α mitotic phosphorylation is essential for cell cycle completion (Chakraborty and Prasanth 2014; Chakraborty et al. 2014). PP1 and PP2a phosphatases dephosphorylate mitotic phosphorylation within the hinge region of HP1α (Chakraborty and Prasanth 2014; Chakraborty et al. 2014; Nishibuchi et al. 2019). Thus, the phosphorylation and dephosphorylation of HP1α’s hinge region regulates key functions of HP1α during the cell cycle to preserve normal segregation of chromosomes.

**Fig. 3:**
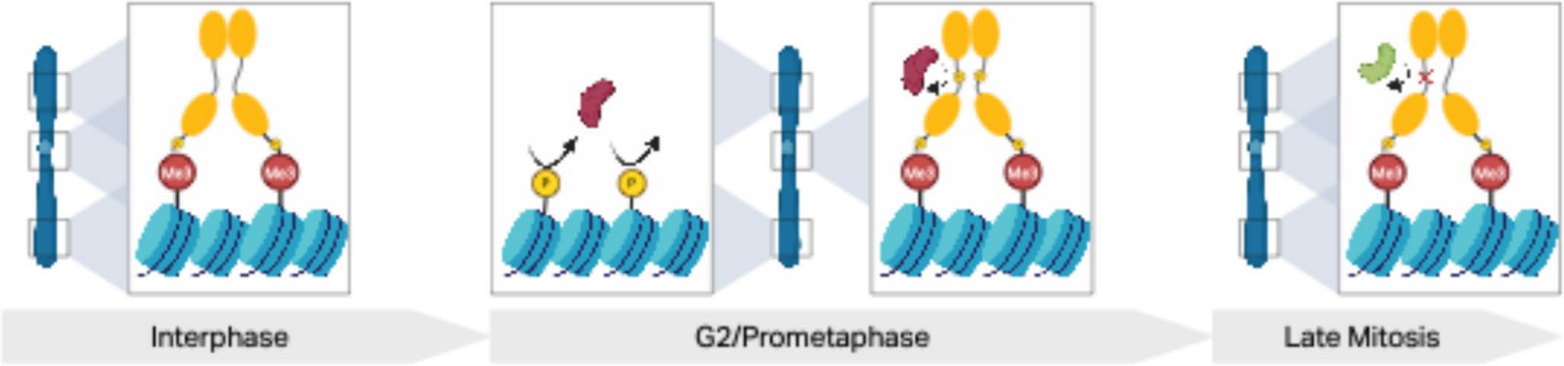
HP1 dynamics during the cell cycle (Illustrated for HP1α). During interphase (left) two isoforms of HP1α (yellow) are present and are constitutively phosphorylated within the NTE to enhance recognition and binding to H3K9me3. In early mitosis (middle) kinases AURKB and NDR1/2 (red) phosphorylate HP1α at S92 and S95, respectively, within the hinge region. This causes a loss of recognition to chromatin and a relocalization to the kinetochores. Additionally, AURKB phosphorylates H3S10, which further removes HP1α’s binding ability to chromatin. Finally, at the end of mitosis phosphatases PP1 and PP2A (green) remove hinge region phosphorylation of HP1α to restore normal functioning of HP1α. Created in BioRender

Together, the work on HP1α from several labs over the past two decades has led to the following model. Serine residues within the hinge region of HP1α are phosphorylated by NDR1/2 and AURKB during G2 and prophase of mitotic division. The phosphorylation achieves three things: (1) it removes HP1α from chromatin by removing its ability to bind to DNA; (2) it relocalizes HP1α to the kinetochore; and (3) it allows association of HP1α to binding partners, such as INCENP, to promote chromosome stability (Minc et al. 1999; Nishibuchi et al. 2019; Williams et al. 2019) (Fig. 3). At the end of mitosis, the same phosphorylation in the HP1α hinge has to be removed to allow HP1α to go back to its interphase localization pattern.

Whether the model for cell-cycle-dependent phosphorylation/dephosphorylation developed for mammalian HP1α applies to other HP1 proteins – either in mammals or in other taxa – is not clear. In *D. melanogaster*, during meiotic division, HP1a behaves like HP1α, and its localization changes in a cell-cycle-dependent manner. HP1a colocalizes with the chromosomal passenger complex (CPC) to promote chromosome-directed spindle assembly (Wang et al. 2021). During prometaphase I, HP1a and the CPC relocate from the chromosome to the central spindle where they then direct biorientation of homologous chromosomes (Wang et al. 2021). After mitosis, HP1a then returns to its typical chromosomal binding pattern, with major enrichment at centromeres and telomeres (Wang et al. 2021). However, in *D. melanogaster*, no data are available regarding the involvement of phosphorylation or other PTMs in these cell-cycle-dependent changes in HP1a enrichment. Thus, the available data highlight the importance of HP1a, and its orthologs for proper chromosomal segregation during cell division. Further studies focusing on HP1 family members other than HP1α in diverse species are needed to determine the extent to which cell-cycle-dependent phosphorylation of HP1 proteins impact cell division and to ascertain if this function is conserved across HP1a orthologs.

## Conclusions and outlook

The HP1 family of proteins play an evolutionarily conserved role in heterochromatin formation, gene regulation, and chromosome segregation. PTMs, particularly phosphorylation, are important for these functions. The ability of HP1 proteins to recognize H3K9 methylation through the CD, dimerize via the CSD, and interact with chromatin and nuclear structures through its hinge region have all been shown to be modulated by phosphorylation, contributing to the multifaceted roles HP1 proteins carry out across eukaryotes. While traditionally characterized as transcriptional repressors and key drivers of heterochromatin spreading, HP1 proteins have broader functions, including transcriptional activation, DNA repair, RNA processing, and most notably, regulation of chromosome behavior during cell division, all of which are impacted by the phosphorylation state of the HP1 protein. In the case of phosphorylation, it dynamically modulates HP1 proteins’ chromatin interactions, nuclear localization, and ability to associate with specific protein partners. The findings from mammalian HP1α are notable, as here phosphorylation in the N-terminal tail enhances binding to repressive H3K9me marks, and phosphorylation within the hinge region, regulated by kinases such as NDR1/2 and AURKB, controls HP1α’s dissociation from chromatin and relocalization to the kinetochore during mitosis. These regulatory events are required for proper chromosome segregation and successful progression through mitosis and meiosis. Importantly, these mechanisms appear conserved among HP1 orthologs such as Swi6 in *S. pombe* and HP1a in *D. melanogaster*, underscoring the evolutionary preservation of this regulation of HP1 proteins by phosphorylation.

**Fig. 4:**
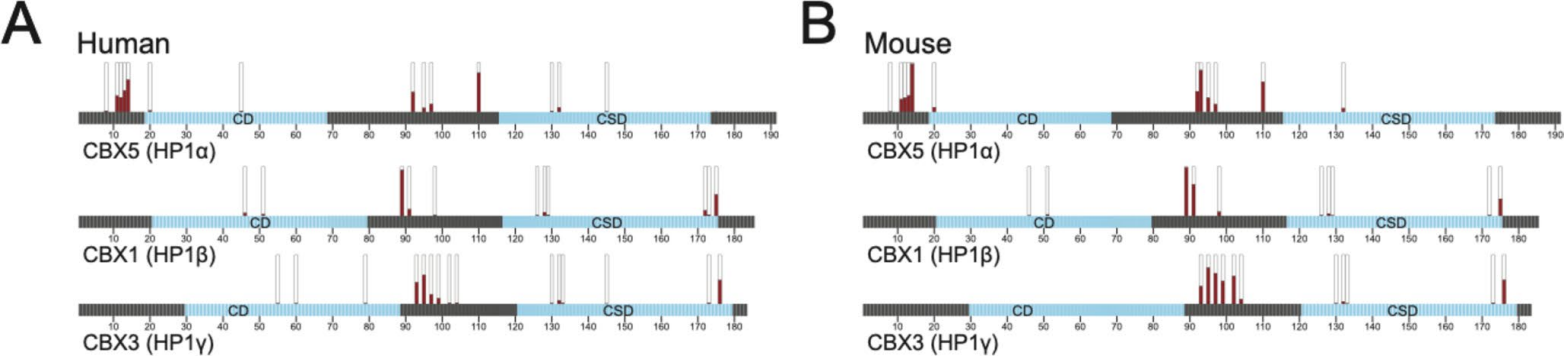
The percent of HP1 molecules phosphorylated varies significantly depending on amino acid residue and HP1 homolog. The percent of molecules phosphorylated at a given amino acid position based on publicly available mass spectrometry data are shown for each of the three mammalian HP1 proteins, HP1α (top), HP1β (middle), and HP1γ (bottom). Stacked bar plots above individual amino acid illustrate the percent of HP1 proteins that are phosphorylated at individual amino acid residues (red), with the percent unphosphorylated shown white. All stacked bar plots add up to 100%. **A**. Data from human samples, extracted from the Human Phospho PeptideAtlas. **B**. Data from mouse samples, extracted from the Mouse Phospho PeptideAtlas

Despite significant advances in our understanding of HP1 proteins, many questions remain. One major question is that of what fraction of HP1 proteins is phosphorylated and at which sites. Given the diverse functions of HP1 proteins, one would expect the fraction of HP1 molecules that are phosphorylated to be different for the various phosphorylation sites and likely vary between tissues, cell cycle phases, and possibly in response to external factors. In studies using 2D electrophoresis or Phos-tag SDS-PAGE gels, it appears that a large fraction of HP1 proteins is phosphorylated (Eissenberg et al. 1994; Zhao et al. 2001; LeRoy et al. 2009; Chakraborty et al. 2014). Mass spectrometry data from large-scale phospho-proteome studies might also shed light on this question, but often, only the number of phosphorylated peptides is reported, omitting information about the unphosphorylated peptides. A hint of what this information might look like can be gained from the Mouse Phospho PeptideAtlas and the Human Phospho PeptideAtlas (Desiere 2006). Querying these atlases for HP1α, HP1β, and HP1γ, one finds that the fraction of molecules being phosphorylated at any given amino acid position varies from less than 1% to over 95% (Fig. 4; Supplemental Table S2). In addition, the fraction of peptides recovered with multiple phosphate groups ranges from 0 to over 50%, illustrating the complexity of the data. These data as well as similar available datasets (Olsen et al. 2006; Huttlin et al. 2010; Ochoa et al. 2020) are an excellent starting point to begin to understand the composition of the cellular pool of HP1 proteins, their phosphorylation status, and how it differs between tissues, cell cycle stages, and in response to stimuli.

Other open questions relate for example to the specific contributions of individual phosphorylation sites to the distinct functions of HP1 proteins, which are still incompletely understood, especially in vivo and for proteins other than HP1α. The interplay between phosphorylation and other PTMs, the coordination of HP1 function with different binding partners, and the role of LLPS in HP1-mediated chromatin dynamics all require further investigation. Further studies of LLPS might reveal if only specific phospho-isoforms of HP1 proteins participate in phase separation, thus potentially providing a switch that allows for compartmentalization of HP1-associated chromatin in some situations but not all. Additionally, it remains to be determined whether mitotic phosphorylation seen in HP1α is conserved in HP1a, Swi6, and HP1 proteins from other taxa, or whether these represent functionally conserved but structurally divergent motifs. Furthermore, as HP1 proteins typically occur in small gene families, studies systematically comparing phosphorylation of the different homologs and their functions are needed.

Together, these available data suggest that phosphorylation of HP1a and its orthologs is a pivotal regulatory mechanism that modulates their activity and impacts a diverse set of chromatin-related functions. Understanding phosphorylation and other PTMs in this important family of chromatin proteins is crucial to fully elucidate how the HP1 protein family contributes to such a broad spectrum of essential cellular processes and achieves multifunctionality.

## Supplementary Information

Below is the link to the electronic supplementary material.

Supplementary Tables 1 & 2 (DOCX 39.3 KB)

## Data Availability

All relevant data and details of resources can be found within the article and its supplementary information.
