# Supplementary material for: Phosphorylation as a regulatory mechanism of HP1 protein multifunctionality
Source: Chromosoma. 2025 Oct 15;134(1):9. doi: 10.1007/s00412-025-00838-0 (PMC12528258; doi:10.1007/s00412-025-00838-0)
Supplement: Supplementary file 1 — Supplementary Tables 1 & 2 (DOCX 39.3 KB) [file 412_2025_838_MOESM1_ESM.docx]

**Supplemental Table 1. Amino acid residues that can be phosphorylated within human HP1α (UniProt: P45973), *D. melanogaster* HP1a (UniProt: P05205), and *S. pombe* Swi6 (UniProt: P40381).** All serine (S), threonine (T), and tyrosine (Y) residue within HP1α/HP1a/Swi6 as well all know kinases (column three) and phosphatases (column 4) are shown.

**A.** Human HP1α (UniProt: P45973)

| \| **Residue #** \| **AA** \| **Kinase** \| **Phosphatase** \| \| --- \| --- \| --- \| --- \| \| 5 \| T \|  \|  \| \| 8 \| T \|  \|  \| \| 11 \| S \| CKII \|  \| \| 12 \| S \| CKII \|  \| \| 13 \| S \| CKII \|  \| \| 14 \| S \| CKII \|  \| \| 20 \| Y \|  \|  \| \| 37 \| Y \|  \|  \| \| 45 \| S \|  \|  \| \| 50 \| T \|  \|  \| \| 64 \| S \|  \|  \| \| 70 \| Y \|  \|  \| \| 85 \| S \|  \|  \| \| 87 \| S \|  \|  \| \| 92 \| S \| AURKB \| PP1/PP2A \| \| 95 \| S \| NDR1/2 \| PP1/PP2A \| \| 97 \| S \|  \|  \| \| 103 \| S \|  \|  \| \| 110 \| S \|  \|  \| \| 130 \| T \|  \|  \| \| 132 \| S \|  \|  \| \| 145 \| T \|  \|  \| \| 167 \| Y \|  \|  \| \| 173 \| T \|  \|  \| \| 177 \| Y \|  \|  \| \| 188 \| T \|  \|  \| \| 191 \| S \|  \|  \| |  |
| --- | --- | --- | --- | --- | --- | --- | --- | --- | --- | --- | --- | --- | --- | --- | --- | --- | --- | --- | --- | --- | --- | --- | --- | --- | --- | --- | --- | --- | --- | --- | --- | --- | --- | --- | --- | --- | --- | --- | --- | --- | --- | --- | --- | --- | --- | --- | --- | --- | --- | --- | --- | --- | --- | --- | --- | --- | --- | --- | --- | --- | --- | --- | --- | --- | --- | --- | --- | --- | --- | --- | --- | --- | --- | --- | --- | --- | --- | --- | --- | --- | --- | --- | --- | --- | --- | --- | --- | --- | --- | --- | --- | --- | --- | --- | --- | --- | --- | --- | --- | --- | --- | --- | --- | --- | --- | --- | --- | --- | --- | --- | --- | --- | --- |

**B.** *D. melanogaster* HP1a (UniProt: P05205)

| \| **Residue #** \| **AA** \| **Kinase** \| **Phosphatase** \| \| --- \| --- \| --- \| --- \| \| 10 \| S \| CKII \|  \| \| 11 \| S \| CKII \|  \| \| 15 \| S \| CKII \|  \| \| 24 \| Y \|  \|  \| \| 41 \| Y \|  \|  \| \| 42 \| Y \|  \|  \| \| 48 \| Y \|  \|  \| \| 51 \| T \|  \|  \| \| 54 \| T \|  \|  \| \| 70 \| Y \|  \|  \| \| 73 \| S \|  \|  \| \| 80 \| S \|  \|  \| \| 83 \| S \|  \|  \| \| 89 \| S \|  \|  \| \| 90 \| S \|  \|  \| \| 91 \| S \|  \|  \| \| 97 \| T \|  \|  \| \| 102 \| S \|  \|  \| \| 103 \| S \|  \|  \| \| 104 \| S \|  \|  \| \| 105 \| T \|  \|  \| \| 106 \| S \|  \|  \| \| 107 \| T \|  \|  \| \| 109 \| S \|  \|  \| \| 113 \| S \|  \|  \| \| 117 \| T \|  \|  \| \| 120 \| S \|  \|  \| \| 124 \| S \|  \|  \| \| 127 \| T \|  \|  \| \| 128 \| T \|  \|  \| \| 134 \| T \|  \|  \| \| 138 \| S \|  \|  \| \| 140 \| S \|  \|  \| \| 141 \| T \|  \|  \| \| 156 \| S \|  \|  \| \| 163 \| T \|  \|  \| \| 179 \| S \|  \|  \| \| 180 \| S \|  \|  \| \| 194 \| Y \|  \|  \| \| 199 \| S \|  \|  \| \| 201 \| Y \|  \|  \| \| 202 \| S \|  \|  \| |
| --- | --- | --- | --- | --- | --- | --- | --- | --- | --- | --- | --- | --- | --- | --- | --- | --- | --- | --- | --- | --- | --- | --- | --- | --- | --- | --- | --- | --- | --- | --- | --- | --- | --- | --- | --- | --- | --- | --- | --- | --- | --- | --- | --- | --- | --- | --- | --- | --- | --- | --- | --- | --- | --- | --- | --- | --- | --- | --- | --- | --- | --- | --- | --- | --- | --- | --- | --- | --- | --- | --- | --- | --- | --- | --- | --- | --- | --- | --- | --- | --- | --- | --- | --- | --- | --- | --- | --- | --- | --- | --- | --- | --- | --- | --- | --- | --- | --- | --- | --- | --- | --- | --- | --- | --- | --- | --- | --- | --- | --- | --- | --- | --- | --- | --- | --- | --- | --- | --- | --- | --- | --- | --- | --- | --- | --- | --- | --- | --- | --- | --- | --- | --- | --- | --- | --- | --- | --- | --- | --- | --- | --- | --- | --- | --- | --- | --- | --- | --- | --- | --- | --- | --- | --- | --- | --- | --- | --- | --- | --- | --- | --- | --- | --- | --- | --- | --- | --- | --- | --- | --- | --- | --- |

**C.** *S. pombe* Swi6 (UniProt: P40381)

| \| **Residue #** \| **AA** \| **Kinase** \| **Phosphatase** \| \| --- \| --- \| --- \| --- \| \| 8 \| S \|  \|  \| \| 9 \| Y \|  \|  \| \| 12 \| S \|  \|  \| \| 13 \| S \|  \|  \| \| 14 \| T \|  \|  \| \| 15 \| S \|  \|  \| \| 18 \| S \| CKII \|  \| \| 24 \| S \| CKII \|  \| \| 30 \| S \|  \|  \| \| 32 \| T \|  \|  \| \| 37 \| S \|  \|  \| \| 43 \| S \|  \|  \| \| 45 \| S \|  \|  \| \| 46 \| S \| CKII \|  \| \| 52 \| S \| CKII \|  \| \| 56 \| S \|  \|  \| \| 58 \| S \|  \|  \| \| 59 \| S \|  \|  \| \| 60 \| S \|  \|  \| \| 81 \| Y \|  \|  \| \| 98 \| Y \|  \|  \| \| 100 \| Y \|  \|  \| \| 107 \| Y \|  \|  \| \| 111 \| S \|  \|  \| \| 114 \| T \|  \|  \| \| 116 \| S \|  \|  \| \| 117 \| S \| CKII \|  \| \| 122 \| S \|  \|  \| \| 131 \| Y \|  \|  \| \| 142 \| S \|  \|  \| \| 147 \| T \|  \|  \| \| 159 \| S \|  \|  \| \| 162 \| S \|  \|  \| \| 165 \| T \|  \|  \| \| 174 \| S \|  \|  \| \| 185 \| T \|  \|  \| \| 192 \| S \|  \|  \| \| 203 \| S \|  \|  \| \| 212 \| S \|  \|  \| \| 216 \| S \|  \|  \| \| 218 \| S \|  \|  \| \| 220 \| S \|  \|  \| \| 224 \| S \|  \|  \| \| 227 \| S \|  \|  \| \| 235 \| T \|  \|  \| \| 237 \| S \|  \|  \| \| 240 \| T \|  \|  \| \| 246 \| S \|  \|  \| \| 259 \| T \|  \|  \| \| 266 \| Y \|  \|  \| \| 268 \| S \|  \|  \| \| 274 \| S \|  \|  \| \| 275 \| S \|  \|  \| \| 278 \| T \|  \|  \| \| 286 \| T \|  \|  \| \| 290 \| Y \|  \|  \| \| 292 \| T \|  \|  \| \| 299 \| S \|  \|  \| \| 303 \| S \|  \|  \| \| 304 \| T \|  \|  \| \| 306 \| T \|  \|  \| \| 318 \| Y \|  \|  \| \| 320 \| S \|  \|  \| \| 323 \| T \|  \|  \| |
| --- | --- | --- | --- | --- | --- | --- | --- | --- | --- | --- | --- | --- | --- | --- | --- | --- | --- | --- | --- | --- | --- | --- | --- | --- | --- | --- | --- | --- | --- | --- | --- | --- | --- | --- | --- | --- | --- | --- | --- | --- | --- | --- | --- | --- | --- | --- | --- | --- | --- | --- | --- | --- | --- | --- | --- | --- | --- | --- | --- | --- | --- | --- | --- | --- | --- | --- | --- | --- | --- | --- | --- | --- | --- | --- | --- | --- | --- | --- | --- | --- | --- | --- | --- | --- | --- | --- | --- | --- | --- | --- | --- | --- | --- | --- | --- | --- | --- | --- | --- | --- | --- | --- | --- | --- | --- | --- | --- | --- | --- | --- | --- | --- | --- | --- | --- | --- | --- | --- | --- | --- | --- | --- | --- | --- | --- | --- | --- | --- | --- | --- | --- | --- | --- | --- | --- | --- | --- | --- | --- | --- | --- | --- | --- | --- | --- | --- | --- | --- | --- | --- | --- | --- | --- | --- | --- | --- | --- | --- | --- | --- | --- | --- | --- | --- | --- | --- | --- | --- | --- | --- | --- | --- | --- | --- | --- | --- | --- | --- | --- | --- | --- | --- | --- | --- | --- | --- | --- | --- | --- | --- | --- | --- | --- | --- | --- | --- | --- | --- | --- | --- | --- | --- | --- | --- | --- | --- | --- | --- | --- | --- | --- | --- | --- | --- | --- | --- | --- | --- | --- | --- | --- | --- | --- | --- | --- | --- | --- | --- | --- | --- | --- | --- | --- | --- | --- | --- | --- | --- | --- | --- | --- | --- | --- | --- | --- | --- | --- | --- | --- | --- | --- | --- | --- | --- | --- | --- | --- | --- | --- | --- |

**Supplemental Table S2. Estimation of the fraction of HP1 proteins that are phosphorylated.**

1. Data for human HP1α, HP1β, and HP1g retrieved from the Human Phospho PeptideAtlas (<https://peptideatlas.org/builds/human/phospho/>)
2. Data for mouse HP1α, HP1β, and HP1g retrieved from the Mouse Phospho PeptideAtlas (<https://peptideatlas.org/builds/mouse/phospho/>)

**A.**

| Species | Protein | Position | % modified | Multiple mods? |
| --- | --- | --- | --- | --- |
| *H. sapiens* | CBX1 (HP1b) | S46 | 5.3 | N |
| *H. sapiens* | CBX1 (HP1b) | T51 | 2.6 | N |
| *H. sapiens* | CBX1 (HP1b) | S89 | 93.2 | Y (7.5%) |
| *H. sapiens* | CBX1 (HP1b) | S91 | 12.8 | Y (7.5%) |
| *H. sapiens* | CBX1 (HP1b) | S98 | 0.8 | Y (7.6%) |
| *H. sapiens* | CBX1 (HP1b) | T126 | 0.5 | N |
| *H. sapiens* | CBX1 (HP1b) | S128 | 7.0 | N |
| *H. sapiens* | CBX1 (HP1b) | S129 | 1.3 | N |
| *H. sapiens* | CBX1 (HP1b) | S172 | 10.5 | Y (0.2%) |
| *H. sapiens* | CBX1 (HP1b) | Y173 | 2.0 | Y (0.2%) |
| *H. sapiens* | CBX1 (HP1b) | S175 | 42.9 | Y (0.2%) |
| *H. sapiens* | CBX3 (HP1g) | T55 | 0.5 | Y (20%) |
| *H. sapiens* | CBX3 (HP1g) | T60 | 0.5 | Y (20%) |
| *H. sapiens* | CBX3 (HP1g) | S79 | 0.5 | Y (20%) |
| *H. sapiens* | CBX3 (HP1g) | S93 | 43.5 | Y (32.8%) |
| *H. sapiens* | CBX3 (HP1g) | S95 | 58.5 | Y (32.5%) |
| *H. sapiens* | CBX3 (HP1g) | S97 | 18.7 | Y (32.5%) |
| *H. sapiens* | CBX3 (HP1g) | S99 | 10.6 | Y (32.5%) |
| *H. sapiens* | CBX3 (HP1g) | S102 | 1.0 | Y (32.5%) |
| *H. sapiens* | CBX3 (HP1g) | S104 | 1.4 | Y(19.0%) |
| *H. sapiens* | CBX3 (HP1g) | T130 | 0.5 | N |
| *H. sapiens* | CBX3 (HP1g) | S132 | 7.0 | N |
| *H. sapiens* | CBX3 (HP1g) | S133 | 1.3 | N |
| *H. sapiens* | CBX3 (HP1g) | S145 | <0.1 | N |
| *H. sapiens* | CBX3 (HP1g) | T173 | 1.5 | Y (1.8%) |
| *H. sapiens* | CBX3 (HP1g) | S176 | 48.0 | Y (1.8%) |
| *H. sapiens* | CBX5 (HP1a) | T8 | 1.8 | Y (47.0%) |
| *H. sapiens* | CBX5 (HP1a) | S11 | 32.4 | Y (47.0%) |
| *H. sapiens* | CBX5 (HP1a) | S12 | 29.3 | Y (47.0%) |
| *H. sapiens* | CBX5 (HP1a) | S13 | 43.3 | Y (47.0%) |
| *H. sapiens* | CBX5 (HP1a) | S14 | 65.3 | Y (46.0%) |
| *H. sapiens* | CBX5 (HP1a) | Y20 | 2.3 | Y (46.0%) |
| *H. sapiens* | CBX5 (HP1a) | S45 | 1.6 | N |
| *H. sapiens* | CBX5 (HP1a) | S92 | 40.0 | Y (2.1%) |
| *H. sapiens* | CBX5 (HP1a) | S95 | 7.4 | Y (2.1%) |
| *H. sapiens* | CBX5 (HP1a) | S97 | 15.3 | Y (2.1%) |
| *H. sapiens* | CBX5 (HP1a) | S110 | 79.5 | N |
| *H. sapiens* | CBX5 (HP1a) | T130 | 1.1 | Y (3.2%) |
| *H. sapiens* | CBX5 (HP1a) | S132 | 8.6 | Y (3.2%) |
| *H. sapiens* | CBX5 (HP1a) | T145 | 0.3 | N |

**B.**

| Species | Protein | Position | % modified | Multiple mods? |
| --- | --- | --- | --- | --- |
| *Mus musculus* | CBX1 (HP1b) | S46 | 0.2 | N |
| *Mus musculus* | CBX1 (HP1b) | T51 | 2.2 | N |
| *Mus musculus* | CBX1 (HP1b) | S89 | 98.2 | Y (7.6%) |
| *Mus musculus* | CBX1 (HP1b) | S91 | 63.7 | Y (7.6%) |
| *Mus musculus* | CBX1 (HP1b) | S98 | 7.7 | Y (7.8%) |
| *Mus musculus* | CBX1 (HP1b) | T126 | 0.5 | N |
| *Mus musculus* | CBX1 (HP1b) | S128 | 4.4 | N |
| *Mus musculus* | CBX1 (HP1b) | Y129 | 0.3 | N |
| *Mus musculus* | CBX1 (HP1b) | Y173 | 0.7 | N |
| *Mus musculus* | CBX1 (HP1b) | S175 | 34.4 | N |
| *Mus musculus* | CBX3 (HP1g) | S93 | 35.5 | Y (40.0%) |
| *Mus musculus* | CBX3 (HP1g) | S95 | 73.8 | Y (40.0%) |
| *Mus musculus* | CBX3 (HP1g) | S97 | 62.2 | Y (40.0%) |
| *Mus musculus* | CBX3 (HP1g) | S99 | 46.1 | Y (40.0%) |
| *Mus musculus* | CBX3 (HP1g) | S102 | 56.5 | Y (40.0%) |
| *Mus musculus* | CBX3 (HP1g) | S104 | 9.3 | Y (1.2%) |
| *Mus musculus* | CBX3 (HP1g) | T130 | 0.5 | N |
| *Mus musculus* | CBX3 (HP1g) | S132 | 4.4 | N |
| *Mus musculus* | CBX3 (HP1g) | S133 | 0.3 | N |
| *Mus musculus* | CBX3 (HP1g) | T173 | 1.6 | Y (3.6%) |
| *Mus musculus* | CBX3 (HP1g) | S176 | 48.5 | Y (3.6%) |
| *Mus musculus* | CBX5 (HP1a) | T8 | 0.3 | Y (54.5%) |
| *Mus musculus* | CBX5 (HP1a) | S11 | 25.6 | Y (54.5%) |
| *Mus musculus* | CBX5 (HP1a) | S12 | 28.7 | Y (54.5%) |
| *Mus musculus* | CBX5 (HP1a) | S13 | 32.9 | Y (54.5%) |
| *Mus musculus* | CBX5 (HP1a) | S14 | 97.7 | Y (49.9%) |
| *Mus musculus* | CBX5 (HP1a) | Y20 | 9.1 | Y (49.9%) |
| *Mus musculus* | CBX5 (HP1a) | S92 | 41.7 | Y (7.3%) |
| *Mus musculus* | CBX5 (HP1a) | S93 | 84.7 | Y (7.3%) |
| *Mus musculus* | CBX5 (HP1a) | S95 | 28.7 | Y (7.3%) |
| *Mus musculus* | CBX5 (HP1a) | S97 | 14.9 | Y (7.3%) |
| *Mus musculus* | CBX5 (HP1a) | S110 | 61.1 | N |
| *Mus musculus* | CBX5 (HP1a) | S132 | 7.1 | N |
